# Mental Healthcare Access among Older Sexual Minority Women with Disabilities

**DOI:** 10.1093/geroni/igaf122.2708

**Published:** 2025-12-31

**Authors:** Jordan Westcott, Matthew Fullen, Alexis Isaac

**Affiliations:** University of Tennessee at Knoxville, Knoxville, Tennessee, United States; Virginia Tech, Blacksburg, Virginia, United States; Virginia Tech, Blacksburg, Virginia, United States

## Abstract

Sexual minority older adults experience health disparities relative to cisgender, straight older adults, including related to mental health and disability (Fredriksen-Goldsen et al., 2013). This may be especially true for older sexual minority women, who experience disparities both relative to the general older adult population and to older sexual minority men (Fredriksen-Goldsen et al., 2017). Despite preliminary evidence of greater mental health needs among this population, little is known about mental healthcare (MHC) access for older sexual minority women with disabilities. Therefore, this study sought to understand how supply- and demand-side factors influenced MHC access among a sample of sexual minority women aged 55 and older who self-identified as having a disability (n = 103). Controlling for mean-centered age, we found that, when supply- and demand-side factors were collapsed into single variables, only supply-side factors predicted MHC access, R2 = .251, F(4, 98) = 8.19, p < .001. In a second analysis focused on unique demand-side factors, we found that availability (b = -.40, p = .015) and accommodation (b = .45, p = .013) of services had significant relationships with MHC access, R2 = .302, F(7, 95) = 5.88, p < .001. Finally, we found that, among demand-side factors, sexual orientation-related healthcare stereotype threat had a significant relationship (b = -.33, p = .002) with MHC access, R2 = .168, F(5, 97) = 3.94, p < .01. Implications for MHC access and service delivery will be discussed.

